# Stone removal in a 5‐year‐old child with extrahepatic biliary obstruction using ERCP: A case report and a mini‐review

**DOI:** 10.1002/ccr3.7620

**Published:** 2023-07-27

**Authors:** Ramin Niknam, Seyede Maryam Mahdavi Mortazavi, Mehdi Ghaderian Jahromi, Marzieh Davoodi, Marzieh Soheili, Maryam Ataollahi, Reza Moshfeghinia

**Affiliations:** ^1^ Gastroenterohepatology Research Center Shiraz University of Medical Sciences Shiraz Iran; ^2^ Pediatric Gastroenterology Fellowship, Department of Pediatrics, School of Medicine Namazi teaching Hospital Shiraz University of Medical Sciences Shiraz Iran; ^3^ Medical Imaging Research Center, Radiology Department Shiraz University of Medical Sciences Shiraz Iran; ^4^ Student Research Committee Shiraz University of Medical Sciences Shiraz Iran; ^5^ College of Pharmacy Western New England University Springfield USA; ^6^ Department of Pediatrics, School of Medicine Namazi teaching Hospital Abu Ali Sina for Medicine & Organ transplant Shiraz University of Medical Sciences Shiraz Iran; ^7^ USERN office Shiraz University of Medical Sciences Shiraz Iran

**Keywords:** common bile duct stones, endoscopic retrograde cholangiopancreatography, hepatobiliary obstruction, left‐sided gallbladder, pediatric

## Abstract

**Key Clinical Message:**

ERCP is a safe and effective method for managing biliary obstruction in children. A case report illustrates successful removal of a bile duct stone in a 5‐year‐old child using ERCP. Pediatric ERCP is a viable option for treating biliary diseases in well‐equipped centers, alongside other approaches.

**Abstract:**

We describe a 5‐year‐old child with extrahepatic biliary stone who successfully underwent endoscopic retrograde cholangiopancreatography for stone removal. He suffered from persistent colicky abdominal pain accompanied by fever that confirmed biliary stone. ERCP along with other methods, can be considered a safe procedure for managing BD in children.

## 1 INTRODUCTION

Biliary obstruction is defined as the blockage of the extrahepatic biliary system.^1^ Causes of extrahepatic biliary blockage include gallstones, cysts, strictures, and neoplasms such as pancreatic cancer, ampullary carcinoma, and cholangiocarcinoma.^2^ Choledocholithiasis from cholesterol stones is the primary biliary obstruction cause in developed countries. Hemolysis, infections, pyogenic cholangiohepatitis, and intrahepatic calculi are more prevalent in Asia. Gallbladder cancer is frequent in specific regions.^2^, ^3^ Choledocholithiasis is rare in children, with most reports focusing on adults. Pediatric cholelithiasis prevalence ranges from 0.13% to 0.3%, but it is higher (2%–6.1%) in obese children and adolescents.^4^

Eighty percent to ninety percent of CBD stone patients can be treated nonsurgically using sphincterotomy, stone extraction with baskets/catheters. For stones >1 cm, procedures like lithotripsy, shock wave lithotripsy, laser lithotripsy, and stenting are used.^4^ Endoscopic retrograde cholangiopancreatography is a diagnostic and therapeutic technique routinely used for adults.^5^ Relative to the published studies regarding adult ERCP, the articles on pediatric ERCP remain limited for several reasons.^6^ Firstly, it is technically more challenging to be used for children. Secondly, pancreaticobiliary pathology in the pediatric population is rare, so the study cannot have an adequate sample size. Additionally, in children weighing more than 10 kg, pediatric ERCP duodenoscopes and accessories have limited application.^7^ Moreover, the advancement of MRCP has limited the use of ERCP for diagnosis.

This case report presents a CBD stone case with a left‐sided gallbladder treated with ERCP in an otherwise healthy 5‐year‐old male. Left‐sided gallbladder refers to a gallbladder located on the left side of the ligamentum teres. It is a rare anomaly usually related to the absence of segment IV, portal vein anomalies, or biliary system anomalies. Diagnosis of the associated anomalies is essential for managing liver transplantation, liver resection, and complicated hepatolithiasis. Preoperative diagnosis of the left‐sided gallbladder with associated anomalies is required to reduce the risks of operative complications.^8^

## 2 CASE REPORT

A 5‐year‐old male was admitted to Namazi hospital (Shiraz, Iran), suffering from a persistent colicky abdominal pain for the last 30 days. Except for his premature birth, he had no history or family history of the following conditions; previous medical problems, taking any medications, past surgeries, abdominal trauma, weight loss, obesity, metabolic syndrome, hemolytic diseases, severe skin itching (Bayler disease), recurrent icterus, anemia, splenectomy, symptoms of chronic liver disease or liver dysfunction, liver disorders (e.g., Wilson's disease), steatorrhea, chronic diarrhea, as well as any underlying causes of gallstone formation. The pain was localized in the epigastric and periumbilical areas exacerbating after consuming dairy and high‐fat foods. The pain was accompanied by fever, vomiting, and constipation. The patient was born with GA = 28 W and a weight of 900 g. His current body weight and height were 16 kg (10–25th percentile) and 112 cm (50–75th percentile), respectively. Vital signs were stable; blood pressure 100/70 mm Hg, heart rate 104 beats/min, respiratory rate 28 breaths/min, and body temperature of 36.5°C. He had hepatomegaly and abdominal tenderness in the epigastric and periumbilical areas in the physical examination.

The hemoglobin, white blood cell, and platelet counts were 12.9 g/dL, 3700/mm3, and 192 × 106/mm^3^, respectively. Hemoglobin electrophoresis was normal. Hemolysis was not noted. Blood chemistries were as following: cholesterol 98 mg/dL (reference range, 120–200 mg/dL), total protein 6 mg/dL (6.1–7.9 mg/dL), albumin 4 mg/dL (3.5–5.6 mg/dL), alkaline phosphatase 962 U/L, AST 104 U/L (15–40 U/L), ALT 180 U/L (5–45 U/L), GGT 160 U/L (5–32 U/L), total bilirubin 1.7 mg/dL (<2.0 mg/dL), amylase 44 U/L (16–91 U/L), and lipase 90 mg/dL (4–29 mg/dL) (Table 1).

**TABLE 1:** Laboratory data during hospitalization.

Laboratory data	On admission	3 days after admission	12 days after admission	4 months later
WBC, /mm^3^	3700	7600		6490
Hb, gm/dL	12/9	12/1		13/9
Platelet, /mm^3^	192,000	467,000		299
PT, sec	14			
INR, index	1/1			
PTT, sec	34			
B.U.N, mg/dL	12			
Cr, mg/dL	0/37			
Na, mEq/L	135			
K, mEq/L	3/7			
AST, IU/L	104	64	25	
ALT, IU/L	180	131	56	12
ALK.P, IU/L	962	1037	665	406
GGT	160	225	114	9/4
Total bilirubin, mg/dL	1/7	1/2		
Direct bilirubin, mg/dL	0/5	0/5		
Total protein, g/dL	6	6/4		
Albumin, g/dL	4	4/2		
Coombs	Neg			
Amylase	44			
Lipase	90			
ESR, mm/h	12			
CRP, mg/L	2			3/6
cholesterol	98			
Triglyceride	122			
Viral marker (HBS Ag, HAV Ig M, and HCV Ab)	Neg			
Urine COPPER	135	437		
IgG	Neg			
Anti‐LKM	Neg			
ANA	Neg			
LDH				363
Uric acid				3/1
Blood culture	No growth			
Urine culture	No growth			

Abdominal ultrasound imaging revealed a distended gallbladder by diffuse wall thickening with a maximum thickness of 3.5 mm, a CBD 10.8 mm in diameter, and an 8.5‐mm‐sized stone in the distal CBD, suggestive of acute choledocholesistitis.

**FIGURE 1:**
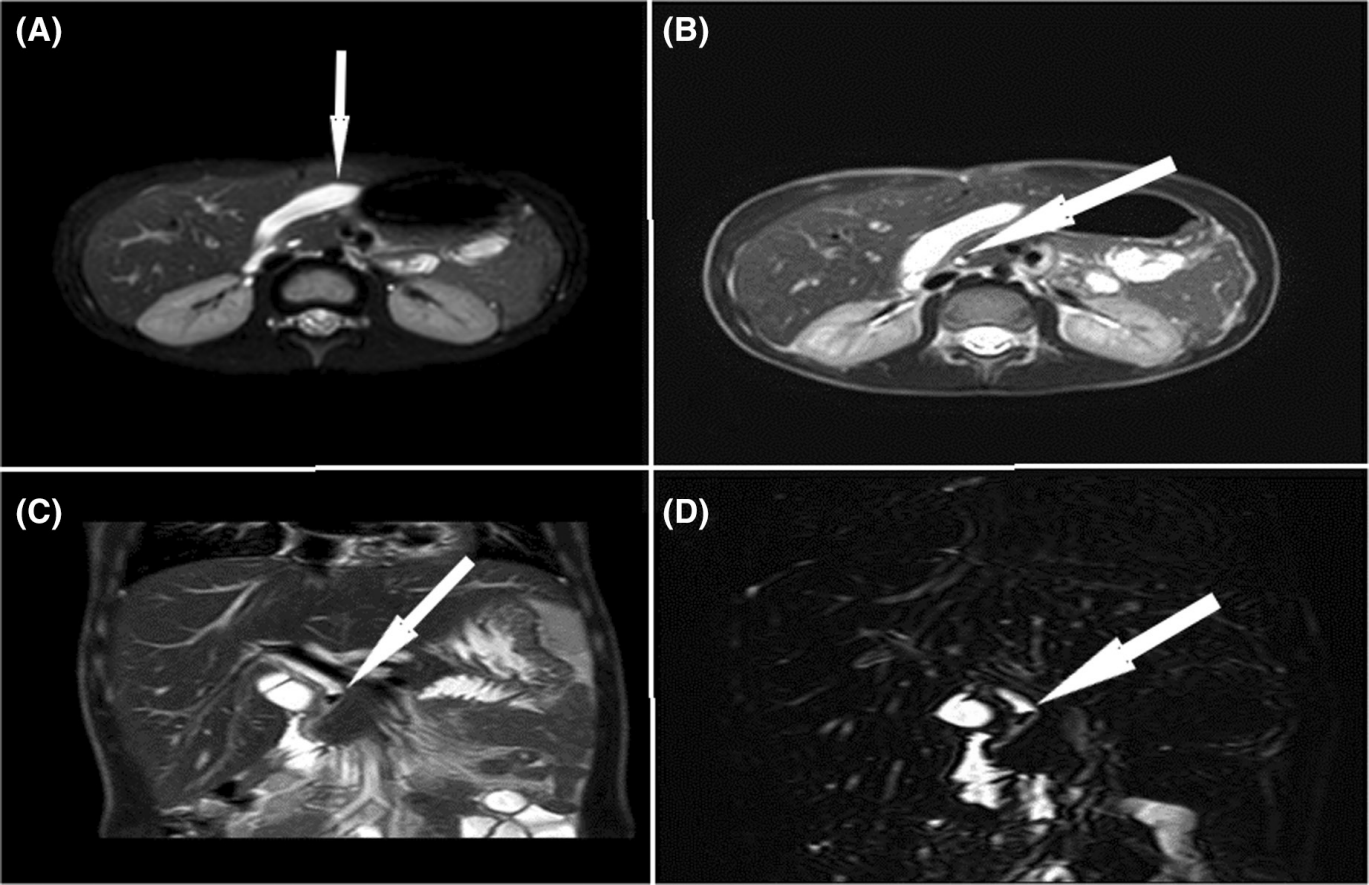
(A) axial T2 MRCP sequence: gallbladder in left subhepatic area. (B) axial T2 MRCP sequence: tiny signal void (dark) stone in the pancreatic portion of CBD. (C) coronal T2 MRCP sequence: signal void tiny stone in the distal part of the CBD. (D) coronal 3D reconstruction MRCP image presenting tiny stone in CBD.

Non per oral diet, intravenous hydration, and administration of analgesics and antibiotics (cefotaxime and metronidazole) were started after the patients' admission. The medical care team decided to perform MRCP and ERCP to evaluate and manage the CBD stone. In MRCP evaluation, mild dilation of central intrahepatic bile ducts and CBD (6 mm) was apparent, associated with a dark signal of a 5 mm stone within the pancreatic portion of CBD located at a 15 mm distance to the major papilla. The gallbladder's position was on the left side of the subhepatic area, concurrent with portal vein abnormality. The main portal vein was trifurcated, and ascending portion (umbilical portion) of the left portal vein was hypoplastic. Additionally, small teres ligament differentiation of segment IV was suboptimal (Figure 1).

**FIGURE 2:**
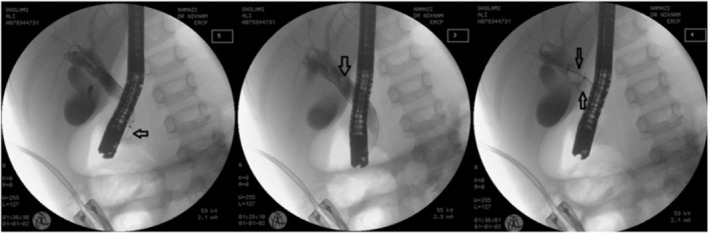
ERCP: dilatation of the CBD with a filling defect (left image) in the middle part and stone removal using a stone retrieval balloon (middle and right images).

**FIGURE 3:**
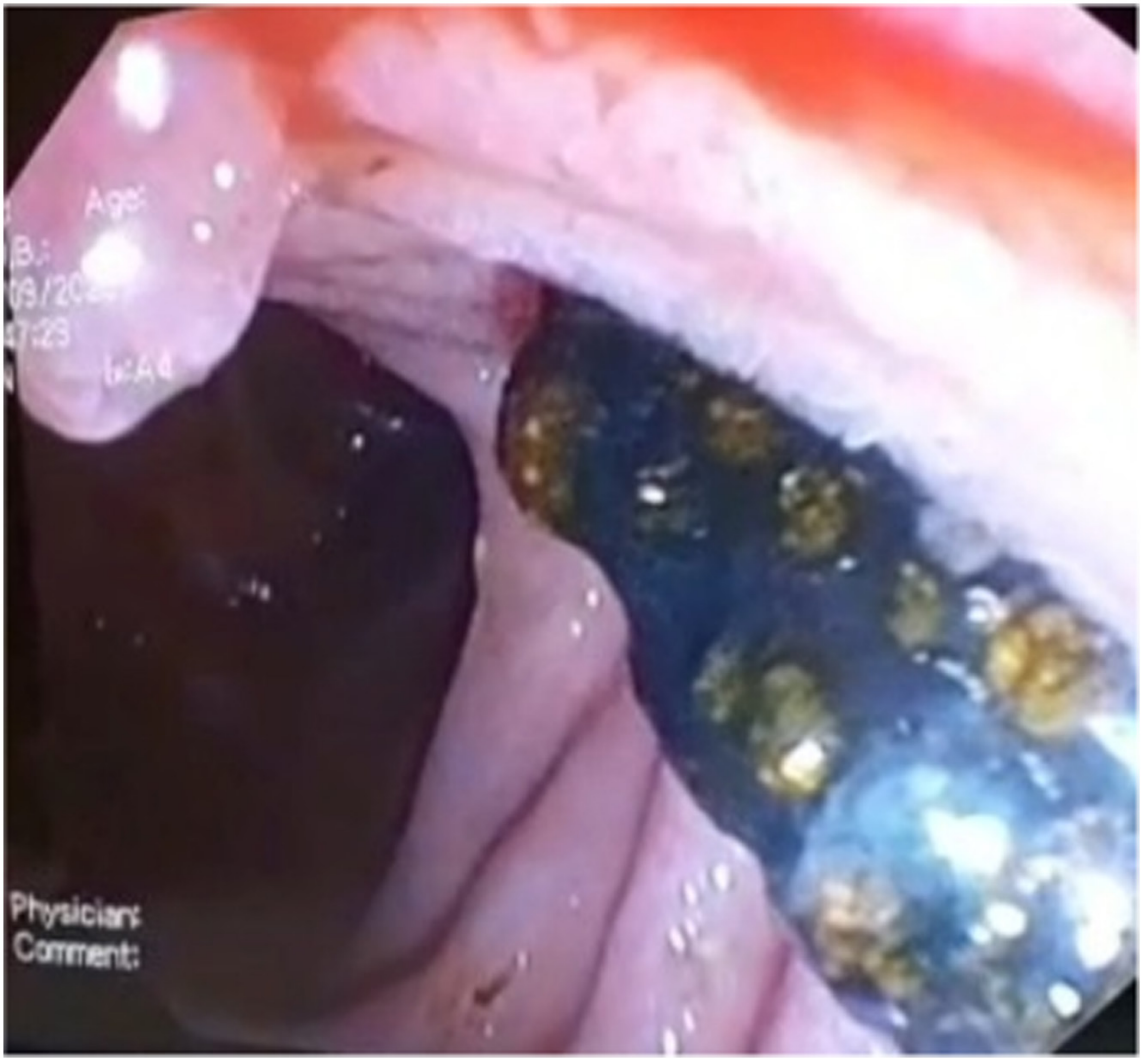
Endoscopic view of pigmented stone after removal using a stone retrieval balloon.

Since our center is well‐equipped, ERCP was performed under general anesthesia. During the procedure, dilatation of CBD with a stone in the middle was investigated (Figure 2). After sphincterotomy, a 10*15 mm pigmented stone was removed using a stone retrieval balloon (Figure 3). After the stone removal, all signs and symptoms were alleviated. There were neither any complications during ERCP nor after discharge. The patient was discharged, and ursodeoxycholic acid (10 mg/kg/dose) and polyethyenglycol syrup (PEG) (1 cc/kg/day) were prescribed for 20 days. No complications, signs, or symptoms were observed in the follow‐up visit, and all laboratory results and abdominal sonography were within the normal range. Finally, the patient was referred for elective cholecystectomy.

## 3 DISCUSSION AND CONCLUSION

CBD stone is a rare condition in children, although it has become more common recently.^7^ Unlike adults, it is mostly symptomatic in children depending on several factors such as age, ethnicity, geographical localization, medical facilities, and referral status.^9^ The most common cause of cholelithiasis in children is a hemolytic disease (20%–30%). Other pathologic situations such as congenital hepatobiliary diseases, obesity, receiving total parenteral nutrition, ileal disease or resection, use of ceftriaxone, metabolic syndrome, choledochal cyst, PFIC, NEC, biliary cirrhosis, prematurity, Wilson disease, cystic fibrosis, congenital heart diseases, and idiopathic cholelithiasis should also be considered as the causes of cholelithiasis in pediatrics.^10^ Houman et al. reported a 12‐year‐old boy with a hemolytic uremic syndrome, established by renal biopsy, who developed cholestatic jaundice. It was discovered by ERCP and extracted by sphincterotomy.^11^ It has been proven that for treating pancreaticobiliary disease, endoscopic sphincterotomy or CBD explorations is a safe method, even in pediatric population.^12^, ^13^ ERCP in pediatric patients is safe and successful, but endoscopists should consider age‐related diseases and lower body weight in young children.^14^ The case presented in this report was symptomatic. The patient had no history or family history of any medical conditions except prematurity. One of the important findings in the MRI and MRCP was the left‐sided gallbladder. Despite stone removal, he is still at risk for CBD stone formation because of having an anomaly in the biliary system. So, the patient was advised to seek cholecystectomy in a soon future.

The left‐sided gallbladder refers to a gallbladder lying on the left side of the falciform ligament.^15^ The reported incidence of this anomaly is estimated to be between 0.1% and 1.2%.^13^ It is very rare and includes three anatomic abnormalities: a right‐sided ligamentum teres, an ectopic left‐sided gallbladder, and a situs inversus.^16^ The possible associated abnormalities with the left‐sided gallbladder are portal vein anomalies, biliary system anomalies, and left lobe hypoplasia.^17^ It has been reported that the likelihood of intraoperative bile duct injuries in individuals with left‐sided gallbladder is higher than the average population (up to 7.3%) due to anomalies of the bile duct, portal vein, and other anatomical structures in the hepatobiliary system.^18^ The patient discussed in our report has trifurcated main portal vein with a hypoplastic ascending portion (umbilical portion) of the left portal vein. Moreover, small teres ligament differentiation of the fourth segment was suboptimal.

As mentioned before, a left‐sided gallbladder is a rare condition. Nevertheless, it is possible to accurately diagnose a left‐sided gallbladder before surgery and perform laparoscopic cholecystectomy by adjusting the port position. Increased size of the left portal vein and distribution of the left portal vein crossing over to the right side of the liver is the crucial common features of the left‐sided gallbladder. These variations probably have considerable clinical implications in managing hepatic resection, including donor hepatectomy.^19^

In recent years, the treatment approaches for managing choledocholithiasis in children have become more specific; however, no gold standard procedure is available yet. The endoscopic approach for managing biliary tract obstruction in medical centers performing ERCP is usually the first choice. Similarly, laparoscopic evaluation of CBD has also proven safe and effective. In the absence of ERCP, laparoscopic investigation can be an appropriate alternative. Laparoscopic cholecystectomy for biliary stone disease in the pediatric population has been well proven as the standard of care, similar to adult patients.^20^ ERCP was commonly applied from 1970 to 1979 to diagnose and treat hepatobiliary diseases in children. However, its use has been more restricted in recent years due to being an invasive procedure.^14^ For instance, Felux et al.^14^ and Lou et al.^21^ have reported that ERCP in children accounts for almost 3.3% and 4% of all ERCP procedures in their centers, respectively. In Asian countries, only a few studies with small sample sizes regarding pediatric ERCP have been performed.^22^ The success rate of endoscopic procedures, especially in children, requires a complete evaluation of the condition before ERCP, and it highly depends on the specialist's skill. It is essential to understand that the ERCP indications should not be extended blindly because they may cause unnecessary complications.^23^

To the best of our knowledge, no report on bile duct stone removal by ERCP in children has been published previously in Iran. This case is the first report of a successful pediatric ERCP for treating a bile duct obstruction due to a stone. The CBD stone was endoscopically removed. ERCP, along with other methods, can be considered a safe procedure for pediatric biliary diseases in well‐equipped centers. The patient is still at risk for CBD stone formation because of having an anomaly in the biliary system. Laparoscopic cholecystectomy is necessary in this patient.

## AUTHOR CONTRIBUTIONS

**Ramin Niknam:** Conceptualization; data curation; visualization; writing – original draft; writing – review and editing. **Seyede Maryam Mahdavi Mortazavi:** Conceptualization; investigation; resources; supervision; writing – original draft. **Mehdi Ghaderian Jahromi:** Project administration; validation; writing – original draft. **Marzieh Davoodi:** Conceptualization; project administration; supervision; validation; visualization; writing – original draft. **Marzieh Soheili:** Project administration; resources; writing – original draft; writing – review and editing. **Maryam Ataollahi:** Conceptualization; data curation; writing – original draft; writing – review and editing. **Reza Moshfeghinia:** Conceptualization; investigation; project administration; supervision; validation; visualization; writing – original draft; writing – review and editing.

## FUNDING INFORMATION

Not applicable.

## CONFLICT OF INTEREST STATEMENT

All authors declare that this manuscript has no conflict of interest.

## ETHICS STATEMENT

This research was approved by the ethics committee of shiraz university of medical sciences with IR.SUMS.REC.1401.665 code.sss.

## CONSENT

Written informed consent was obtained from the patient's mother to publish this case report and any accompanying images. All authors have viewed and agreed to the submission.

## Data Availability

The data supporting this case report's findings is available from the corresponding author upon request.
